# The Role of L-Selectin in HIV Infection

**DOI:** 10.3389/fmicb.2021.725741

**Published:** 2021-09-29

**Authors:** Jason Segura, Biao He, Joanna Ireland, Zhongcheng Zou, Thomas Shen, Gwynne Roth, Peter D. Sun

**Affiliations:** Laboratory of Immunogenetics, National Institute of Allergy and Infectious Diseases, National Institutes of Health, Rockville, MD, United States

**Keywords:** L-selectin (CD62L), HIV-1 infection, envelope gp120, ADAM metalloproteinases, shedding, viral release, viral entry

## Abstract

HIV envelope glycoprotein is the most heavily glycosylated viral protein complex identified with over 20 glycans on its surface. This glycan canopy is thought to primarily shield the virus from host immune recognition as glycans are poor immunogens in general, however rare HIV neutralizing antibodies nevertheless potently recognize the glycan epitopes. While CD4 and chemokine receptors have been known as viral entry receptor and coreceptor, for many years the role of viral glycans in HIV entry was controversial. Recently, we showed that HIV envelope glycan binds to L-selectin in solution and on CD4 T lymphocytes. The viral glycan and L-selectin interaction functions to facilitate the viral adhesion and entry. Upon entry, infected CD4 T lymphocytes are stimulated to progressively shed L-selectin and suppressing this lectin receptor shedding greatly reduced HIV viral release and caused aggregation of diminutive virus-like particles within experimental infections and from infected primary T lymphocytes derived from both viremic and aviremic individuals. As shedding of L-selectin is mediated by ADAM metalloproteinases downstream of host-cell stimulation, these findings showed a novel mechanism for HIV viral release and offer a potential new class of anti-HIV compounds.

## Introduction

Many phases of the HIV lifecycle, including the viral entry, reverse transcription and integration, viral gene transcription, translation and replication, and viral release and maturation, have been intensely studied over the years and targeted by the development of highly active antiretroviral therapies (HAART/ART; Deeks et al., 2015). The success of combinatory ART (cART) has ushered in an era searching for a functionally cured state (Saag et al., 2020). To date, there are four classes of FDA-approved antiviral inhibitors targeting distinct phases of the viral lifecycle for frontline and prolonged suppression of infection, with generational advances in efficacy and resistance barrier: entry/fusion inhibitors (Matthews et al., 2004; Tsibris and Kuritzkes, 2007; Emu et al., 2018), nucleoside/non-nucleoside reverse transcriptase inhibitors (NRTI/NNRTI; Holec et al., 2017), integrase inhibitors (INTI; Lusic and Siliciano, 2017), and protease inhibitors (Flexner, 1998; Paton et al., 2015). Notably, while there is a growing development of inhibitors that target viral transcription (Pinto et al., 2019; Yeh et al., 2020), there are no FDA-approved therapeutics targeting viral release. Recent advances towards prolonging suppression of viremia have revealed late-stage capsid assembly perturbation by a small molecule inhibitor suggesting success is possible with targeting late mechanisms crucial to viral budding (Link et al., 2020).

Investigations into the mechanisms acting on HIV release have revealed a complexing matrix of viral strategies that counter host-cell restriction factors to facilitate the successful trafficking of viral components that assemble and release at the plasma membrane (Ramdas et al., 2020; Rose et al., 2020). For most part, HIV release from either productive infected cells or the latent reservoir is thought to be spontaneous, but can be modulated by various host-cell restrictions, including CD317/BST-2/tetherin (Neil et al., 2008), TIM family membrane proteins (Li et al., 2014), serine incorporator membrane-spanning proteins (SERINC; Usami et al., 2015). While these restriction factors exhibited clear antiviral effect in HIV susceptible cell lines, some of them, including BST-2/tetherin and TIM members are not well expressed in primary CD4 T cells and it is difficult to develop compounds enhancing the expression of these restriction factors. In addition, the viral encoded nef and vpu have been shown to antagonize host restriction factors by actively promoting their degradations (Neil et al., 2008; Jolly et al., 2010). More recently, L-selectin/CD62L has been identified as an HIV adhesion receptor (Kononchik et al., 2018). Like BST-2/tetherin, L-selectin expressions are regulated by interferon-dependent processes during host-cell inflammation for several pathogens and disease states (Wang et al., 2010; Yang et al., 2011; Arias and Evans, 2014). Interestingly, L-selectin shedding appears required for HIV release from infected cells (Kononchik et al., 2018). Unlike tetherin and TIM members, however, L-selectin is abundantly expressed on primary CD4 T cells, and inhibition of L-selectin shedding presents a more efficacious target for suppressing the viral infection. These recent findings also showed that HIV release is not spontaneous and revealed a potential strategy to suppress HIV release from latently infected cellular reservoirs.

HIV envelope gp120 binds many lectins, DC-SIGN, Siglecs, and carbohydrate-binding Cyanovirin-N (CVN; Curtis et al., 1992; Mori et al., 1998; Esser et al., 1999; Snyder et al., 2005; Zou et al., 2011). Binding to DC-SIGN captures HIV by dendritic cells while binding to Siglec-1 facilitates HIV infection of macrophage (Snyder et al., 2005; Zou et al., 2011). Selectin family consists of L-selectin (CD62L), E-selectin and P-selectin and they are named according to their main cell origin with L-selectin present on leukocytes, E-selectin on activated endothelial cells, and P-selectin on activated platelets and endothelial cells (Tedder et al., 1995a). L-selectin (CD62L) functions to provide leukocyte rolling adhesion on endothelial cells (Gallatin et al., 1983; Berg et al., 1993), thus, regulates the migration of leukocytes to peripheral lymph nodes and sites of inflammation (Tedder et al., 1995b). It is one of the earliest surface markers on lymphoid-primed hematopoietic stem cell and is constitutively expressed on most circulating leucocytes (Alon et al., 1997; Ivetic et al., 2019). L-selectin consists of a C-type lectin domain, EGF-like domain, sushi domain, transmembrane domain and cytoplasmic tail (Spencer et al., 2017). The C-type lectin domain interacts with numerous glycans which is involved in rolling adhesion between leucocytes and endothelial cells (Fuhlbrigge et al., 1996). The cleavage of L-selectin can be induced by cell activation agonists such as PMA or infection (Kahn et al., 1994; Kononchik et al., 2018).

For HIV infection, the regulation of L-selectin shedding is especially meaningful. The paradoxical function of L-selectin in HIV biology, the one promotes viral adhesion to facilitate host cell entry vs. and the one hinders viral release through virion tethering, revealed complex roles of L-selectin in HIV lifecycle. Both are based on the same biochemical interaction, the binding of glycosylated envelope protein to cell surface L-selectin on CD4 T cells. As a consequence, a beneficial interaction to facilitate the adhesion step in viral entry of a multi-round infection becomes detrimental to successive viral dissemination that HIV induces the shedding of L-selectin to permit viral dissemination.

### Cellular Ligands of L-Selectin

L-selectin was found essential for homing of naive lymphocytes to secondary lymphoid organs in carbohydrate dependent manner (Gesner and Ginsburg, 1964; Butcher and Picker, 1996). The ligand of L-selectin on high endothelial venules (HEV) are often O-linked Sialyl-Lewis X type sulfated glycans that can be blocked by MECA-79, an antibody specific for 6-sulfo sialyl-Lewis X in a sulfation and sialic acid dependent manner (Foxall et al., 1992; Mitsuoka et al., 1998). The O-linked sulfated sialyl-Lewis x was found on peripheral lymph node, CD34, glycosylation-dependent cell adhesion molecule (GlyCAM-1) and mucosal vascular addressin cell adhesion molecule-1 (MAdCAM-1) on HEV and all were identified as L-selectin ligands (Streeter et al., 1988; Baumheter et al., 1993; Berg et al., 1993; Puri et al., 1995). Further, sulfated glycans can be induced on HEV-like blood vessels at the site of inflammation caused by ulcerative colitis, rheumatoid arthritis or Helicobacter pylor infection (Kobayashi et al., 2004). Ligands of L-selectin also include abluminal and extravascular glycoproteins, such as heparin sulfate proteoglycan (Rosen, 2004). In general, the binding of L-selectin to its ligands mediates low affinity rolling adhesion of lymphocytes along HEV prior to high affinity attachment mediated by the interaction between LFA-1 on lymphocytes and ICAMs on HEV (Xu et al., 2003). L-selectin binding initiates the activation of integrins (Lawrence and Springer, 1991), activates chemokine receptors to promote trans-endothelial migration (Ding et al., 2003). However, mice lacking O-linked oligosaccharide still had considerable lymphocyte-homing activity and the remaining L-selectin attachment was abolished after enzymatic removal of N-glycans attached to HEV and CD34 (Mitoma et al., 2007). Therefore, both O- and N-glycans are ligands of L-selectin.

### HIV Infections Regulate L-Selectin Expression

As L-selectin plays important roles in lymphocyte and leukocyte adhesion, activation and homing, as well as serves as a marker for central memory T cells, its expression is therefore often used as an indicator for HIV infection caused immune activation and dysregulation. Early clinical observations from HIV infected individuals showed lower L-selectin expression on T cells and neutrophils compared to healthy controls or individuals on ART therapy, suggesting the viral infection caused protracted immune activation and dysregulated lymphocyte homing (Moore et al., 1998; Gainet et al., 1999; Meddows-Taylor et al., 2001; Schneider-Hohendorf et al., 2014). This is further supported by the observation that antiviral therapy restored L-selectin expression in HIV infected, ART-naïve individuals (Vassena et al., 2016). The soluble L-selectin levels detected in circulation were found to be higher in infected than healthy individuals (Kourtis et al., 2000, 2003; Meddows-Taylor et al., 2001; Schramm et al., 2007; Yang et al., 2014), reminisce to elevated soluble selectin found in autoimmune diseases, including rheumatoid arthritis, systemic sclerosis, and systemic lupus erythematosus (Sfikakis et al., 1999; Shimada et al., 2001; Ates et al., 2004). These early studies established the dynamics of HIV infection in overall T lymphocyte and neutrophil activations and the infection resulted dysregulation in immune functions. Many of these studies, however, did not address any specific mechanism involving L-selectin in HIV biology.

In human T cells, the expression of L-selectin appears to be controlled by members of Forkhead box transcription factors, such as FOXO1 (Fabre et al., 2008). Suppression of FOXO1 has been implicated in HIV infection-mediated downregulation of L-selectin expression in infected CD4 T cells (Trinite et al., 2014). Early mechanistic work showed that mere binding of viral envelope gp120 protein to CD4 and CXCR4 was sufficient to induce down regulation in L-selectin expression (Marschner et al., 1999; Wang et al., 2004). The envelope binding, however, was found insufficient by Trinite et al. (2014) and L-selectin down regulation required HIV infection and was mediated by the suppression of transcription factor Foxo1 and KLF2. Vassena et al. (2015) showed that HIV nef and vpu contributed to the viral induced L-selectin down regulation that was attributed to the retention of the receptor in perinuclear compartments as a result of nef association. Interestingly, HIV encoded vpr appears to induce L-selectin transcription and counter the nef and vpu-mediated receptor downregulation (Giuliani et al., 2018). These publications established specific viral-induced cellular signaling changes in infected cells, thus providing a molecular mechanism for HIV infection regulated host immune functions, including T cell homing to site of inflammation and viral evasion to immune response. Previously, L-selectin shedding during HIV infections was also reported with the assumption that the shedding of L-selectin helps HIV to evade immune detection (Wang et al., 2004; Vassena et al., 2016). In addition, the work of Kononchik et al. (2018) also supports an HIV infection-induced shedding of L-selectin as another mechanism to down regulate the selectin expression on infected cells. Thus, HIV infection induces both L-selectin shedding and intracellular retention. While both shedding and retention are likely connected to cellular signaling apparatus, it is not clear to what extend they overlap, for example, are linked by common cellular signaling pathways, such as PI3K signaling pathway (Trinite et al., 2014).

### Binding of Gp120 To L-Selectin Enhanced HIV Viral Entry

Compared to its cellular ligands, little is known about L-selectin recognition of viral ligands. HIV-1 envelope is heavily glycosylated with over 20N-linked glycans on each envelope monomer. In general, L-selectin prefers O-linked glycans with few exceptions in which N-glycans are linked to 6-sulfo sialy Lewis X (Clark et al., 1998; Mitoma et al., 2007). However, the densely populated gp120 glycans may enhance the avidity of L-selectin binding as soluble glycosylated but not deglycosylated gp120 readily bound to L-selectin with 50-300nM affinities (Kononchik et al., 2018). L-selectin binding to gp120 exhibited typical C-type lectin receptor characteristics and can be inhibited with EDTA and various competing carbohydrates, including heparin, fucoidan and sialyl-Lewis X. In addition, cell surface expressed L-selectin can bind gp120 and capture HIV virions.

**Figure 1:**
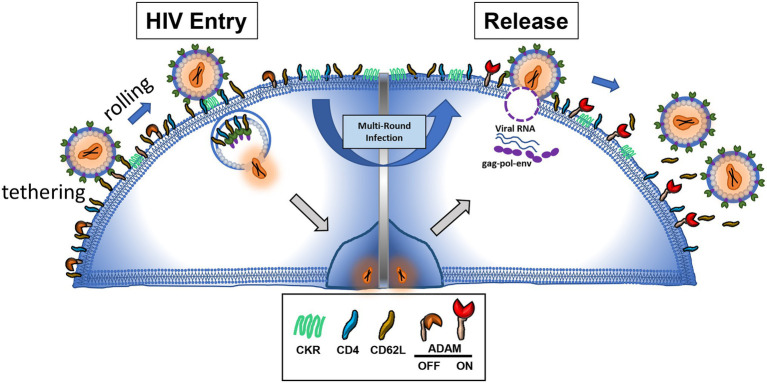
HIV envelope binding to L-selectin facilitates HIV adhesion and infection to CD4 T cells (**left**). The same envelope and L-selectin interaction hinders the release of budding virions (**right**).

The binding of viral envelope glycan to L-selectin provided viral adhesion to target cells. Overexpression of L-selectin in CEM T cells enhanced HIV infection while knockdown of the gene decreased the infection. Consistently, HIV viruses produced in GnTI^−^ 293T cells that are deficient in mature complex N-glycans infected CD8-depleted peripheral blood mononuclear cells (PBMC) less than their glycan sufficient counterparts. These results support the notion that binding of gp120 to L-selectin enhanced HIV infection in a glycan dependent way. Mechanistically, the binding of HIV envelope glycan to L-selectin may provide the rolling adhesion for the virion on CD4+ T lymphocytes, thus facilitate the binding of HIV to CD4 and other coreceptors (Figure 1). It is also possible that L-selectin binding initiates a conformational change to facilitate the envelope binding to CD4 (Wang et al., 2020).

### L-Selectin Shedding Facilitates HIV Viral Release

L-selectin can be cleaved at its membrane proximal region from cell surface by a disintegrin and metalloproteinase domain-containing proteins, ADAM10 and ADAM17, in response to inflammation or apoptotic signals (Wang et al., 2010). Shedding of L-selectin is part of normal host immune response and regulates migration of neutrophils and T cells in and out of the sites of inflammation (Kishimoto et al., 1989; Galkina et al., 2003). Excessive shedding of L-selectin is observed to correlate with the severity of lupus erythematosus (SLE) and type I diabetes (Font et al., 2000; Kretowski et al., 2000).

**Figure 2:**
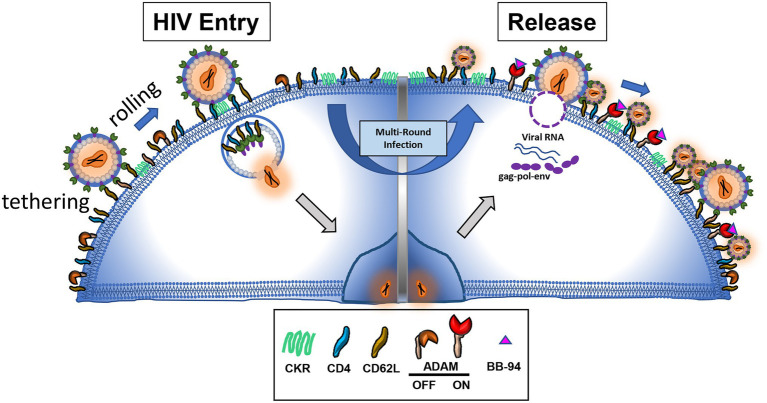
L-selectin shedding is inhibited by metalloproteinase inhibitor BB-94, resulting in tethered and defective virions on infected cells.

Early patient studies showed increased soluble L-selectin levels detected in HIV infected serum samples compared to healthy controls (Kourtis et al., 2000, 2003; Meddows-Taylor et al., 2001; Schramm et al., 2007; Yang et al., 2014). This HIV infection associated increase in soluble L-selectin, however, was mechanistically associated with dysregulated cytokine production and immune exhaustion rather than direct viral induced shedding. In experimental HIV infections, the loss of the CD4^+^/CD62L^+^ and the gain of the CD4^−^/CD62L^−^ lymphocytes are apparent (Kononchik et al., 2018). The progressive loss of L-selectin expression in infected CD4^+^ T cells correlated with the viral infection suggesting that the shedding of L-selectin is viral infection-induced. HIV infection induced down regulation of entry receptors (Garcia and Miller, 1991; Alkhatib, 2009). The downregulation of CD4 expression through envelope gp120-mediated internalization on infected cells was thought to prevent superinfection, more than one virus entering the same host cell, a strategy to maximize viral transmission, while the down regulation of L-selectin may be part of viral evasion to immune response. Since L-selectin facilitates HIV adhesion and infection of CD4 T cells, inhibition of its shedding was predicted to enhance the viral infection, presumably increasing the mode of superinfection (Garcia and Miller, 1991; Michel et al., 2005). When metalloproteinase activity was inhibited, infected lymphocytes retained L-selectin expressions. HIV infection, however, was suppressed in the presence of metalloproteinase inhibitors. Further experiments showed that the metalloproteinase inhibitors did not affect the viral entry but hampered the viral release, resulting in tethering of budding virions on cell surface. These cell surface tethered infectious virions can be recovered by trypsinization (Kononchik et al., 2018). Strikingly, many tethered virion-like particles exhibit diminutive morphology in electron microscopy images in the presence of L-selectin shedding inhibitors. These data support the notion that HIV viral release from infected CD4 T cells requires shedding of L-selectin (Figure 2). Consistently, inhibition of L-selectin shedding also suppressed HIV release from viral reservoir CD4 T cells derived from infected individuals (Kononchik et al., 2018). While new to HIV infection, the concept of shedding of viral attachment receptor to facilitate viral release is known to influenza infections, in which the viral attachment receptor, sialic acid, is cleaved by viral neuraminidase to facilitate the viral release. Notably, most FDA approved drugs for influenza infections are neuraminidase inhibitors, suggesting viral release is a good target for developing antiviral compounds.

### Viral Regulation of L-Selectin Shedding

The cellular signaling pathways controlling L-selectin expression and shedding has not been fully characterized. The shedding of L-selectin has been linked to inflammation and apoptotic activations (Wang et al., 2010). It involves caspase activation of ADAM10,17 metalloproteinases through phosphatidylserine exposure (Sommer et al., 2016). The mechanism of HIV induced L-selectin shedding is less clear. Earlier work showed that ligation of CD4 and chemokine receptor CXCR4 by HIV envelope gp120 induced L-selectin shedding (Marschner et al., 1999; Wang et al., 2004). Nef and vpu are likely the viral genes to regulate L-selectin shedding as they have been implicated in previous studies investigating viral induced inflammatory dysregulation of cell surface markers. Nef is an accessory protein required for viral transmission in primary cells and for disease progression in humans and animal models (Sodroski et al., 1986; Strebel et al., 1987; Kestler et al., 1991; Gulizia et al., 1997; Rhodes et al., 2000; Chakrabarti et al., 2003). Further, nef has been shown to activate host cellular signaling pathways, such as PKC complex, resulting in downregulation of CD4 expressions (Smith et al., 1996; Rasola et al., 2001; Wolf et al., 2008; Dikeakos et al., 2012; Pereira and daSilva, 2016; Jacob et al., 2017). Nef has also been linked to cellular apoptosis (Jacob et al., 2017), and the activation of ADAM10 and 17 through paxillin (Lee et al., 2013). Vpu is linked to HIV release through antagonizing tetherin (Neil et al., 2008). It is likely that these known viral-host interactions somehow form a coordinated signaling pathway leading to the activation of L-selectin shedding and viral release.

Interestingly, both P-selectin glycoprotein ligand 1 (PSGL-1) and CD43, have been recently reported to be incorporated into HIV virion and inhibit the virion attachment to CD4 T cells (Liu et al., 2019; Murakami et al., 2020). Both PSGL-1 and CD43 were found associated with virions and are thought to interfere viral envelope binding to CD4 and chemokine receptors due to a non-specific size preclusion (Fu et al., 2020; Murakami et al., 2020). PSGL-1 exists in a glycosylated mucin-like homodimer of 240kD protein, approximately half the size of an HIV envelope trimer. CD43, also known as sialophorin, is also heavily glycosylated with an apparent molecular weight of ~140kD. Both PSGL-1 and CD43 appear to be smaller than HIV envelope gp160. However, both are heavily glycosylated. In fact, PSGL-1 is a known ligand of L-, E- and P-selectin. This brings a possibility, in addition to the proposed interference by the size of PSGL-1, that the binding between virion-expressed PSGL-1 and host L-selectin prevents the dissemination of virus. It is conceivable that the virion-expressed PSGL-1 competitively inhibits HIV envelope binding to L-selectin on CD4 T cells, resulting in none-productive viral adhesion but not entry. If so, gp120 binding to L-selectin not only functions to promote viral adhesion, it is also a prerequisite for the viral envelope binding to CD4 and chemokine receptor. It is worth noting that both L-selectin and PSGL-1 are markers of inflammation, and both expressions are downregulated during acute infections (Kononchik et al., 2018; Fu et al., 2020). It is conceivable that the viral infection-induced downregulation of both L-selectin and PSGL-1 expressions on infected cells facilitates viral dissemination. While L-selectin downregulation is through shedding, the downregulation of PSGL-1 involved Vpu-induced ubiquitination and degradation pathway that may be targeted for antiviral development (Liu et al., 2019; Fu et al., 2020). Taken together, the growing role of selectins in viral pathogenesis and host cell defense requires further investigation into the transcriptomic state of infected cells that regulate upstream pathways leading to the currently identified viral restriction mechanisms.

### Conclusion and Future Perspective

L-selectin not only regulates the migration of leucocytes but also functions as a receptor for HIV adhesion to CD4 T lymphocytes and facilitates the viral entry. Upon viral entry, infected T cells loose L-selectin expression through both receptor internalization and shedding by ADAM metalloproteinases and the inhibition of L-selectin shedding resulted in budding virions aggregation, impaired the viral release in experimental infections and reduced viral RNA released from ART-suppressed viral reservoirs (Kononchik et al., 2018). It is likely that both L-selectin internalization and shedding occur at different stages of HIV infections. For example, attachment of HIV virus may induce internalization of viral envelope bound L-selectin to endosomal compartments during viral entry. Such internalization serves to initiate cellular signaling through interaction with nef and other viral proteins, leading to the activation of kinases and transcription machinery for viral replication. The infection induced cellular activation is not only required for viral replication, but also needed for the shedding of remaining L-selectin on infected T cells to facilitate the viral release. These recent findings suggest that the regulation of L-selectin is a promising target for developing anti-HIV therapies. While the expression and shedding of L-selectin play important roles in HIV biology, many questions remain to be addressed. The structural recognition of L-selectin to HIV gp120 glycans remains unresolved. As the intrinsic carbohydrate binding affinity of L-selectin is low (Klopocki et al., 2008), it is likely that L-selectin and gp120 binding is enhanced by the avidity of multiple glycans distributed on the envelope protein. Secondly, L-selectin is also expressed on macrophages, a known viral reservoir. Like T cells, macrophages also regulate L-selectin shedding by host cell metalloproteinases (Tedder et al., 1995a; Link et al., 2017; Wong et al., 2019). It remains to be seen if HIV release from infected macrophages also requires shedding of L-selectin. Third, L-selectin, as a member of C-type lectin receptor, presumably recognize viral glycans independent of their peptide sequences. Namely, the effect of L-selectin on HIV entry and release may be generalized to other lectin receptors interacting with viruses with heavily glycosylated envelopes.

## Author Contributions

JS, JI, and ZZ did the experiments for the original publications. JS, BH, and PS wrote the manuscript. TS and GR contributed to the write-up. All authors contributed to the article and approved the submitted version.

## Funding

This work was supported in part by the National Institutes of Health Strategic Fund in HIV/AIDS research from Office of AIDS Research, and by the Intramural Research Program of National Institute of Allergy and Infectious Diseases, National Institutes of Health.

## Conflict of Interest

The authors declare that the research was conducted in the absence of any commercial or financial relationships that could be construed as a potential conflict of interest.

## Publisher’s Note

All claims expressed in this article are solely those of the authors and do not necessarily represent those of their affiliated organizations, or those of the publisher, the editors and the reviewers. Any product that may be evaluated in this article, or claim that may be made by its manufacturer, is not guaranteed or endorsed by the publisher.
